# The effect of Immersive Virtual Reality on balance: an exploratory study on the feasibility of head-mounted displays for balance evaluation

**DOI:** 10.1038/s41598-024-54274-8

**Published:** 2024-02-12

**Authors:** Oskar Rosiak, Nikodem Pietrzak, Anna Szczęsna, Izabela Kulczak, Grzegorz Zwoliński, Dorota Kamińska, Wiesław Konopka, Magdalena Jozefowicz-Korczynska

**Affiliations:** 1 Department of Otolaryngology, Polish Mother’s Memorial Hospital Research Institute, Lodz, Poland; 2 Department of Otolaryngology, Student’s Scientific Circle, Polish Mother’s Memorial Hospital Research Institute, Lodz, Poland; 3 Institute of Mechatronics and Information Systems, Lodz University of Technology, Lodz, Poland; 4 Balance Disorders Unit, Department of Otolaryngology, Medical University of Lodz, The Norbert Barlicki Memorial Teaching Hospital, Lodz, Poland

**Keywords:** Diagnosis, Preclinical research

## Abstract

Global interest in applying virtual reality (VR) in research and medicine has grown significantly, with potential benefits for patients suffering from balance disorders, instability, and a high risk of falling. This exploratory study assesses the impact of immersive VR (IVR) delivered through a head-mounted display (HMD) on balance and explores the feasibility of using the HMD VR unit as a standalone posturography tool. Using the Meta Quest 2 HMD and a mid-range Android smartphone equipped with standard sensors, the research employed a VR environment that simulated a ship at sea, with thirty-eight healthy participants with no otoneurologic abnormalities. Measurements were conducted in repeated trials, including static assessments on both stable ground and foam, as well as a 3-m walk. This was conducted in two settings: one within a VR environment with three different intensity levels and the other in non-VR settings. Statistical analysis and clinical evaluation revealed that IVR with HMD influences head-level sway velocity, which correlates with increased visual disturbance, suggesting its potential as a low-risk standalone posturography tool.

## Introduction

The application of virtual reality (VR) in research and medicine is gaining significant attention worldwide. An astonishing 2928 VR-related articles were added to the PubMed database in 2021, in contrast to 2010, when only 418 were added. VR is nowadays applied in many aspects of medicine, such as training surgeons^1^ or treating psychological and physiological disorders. In medicine, one of the principal areas of study on VR application is the rehabilitation of patients. The studies are so numerous that several meta-analyses have already been published measuring the effects of VR interventions. To date, VR has been studied thoroughly in the rehabilitation of stroke, both in cognitive^2^ and motor skills domains^3,4^, dementia^5^, motor function recovery in spinal cord injury^6^, and post-traumatic stress disorder^7^. Studies have also been conducted in the pediatric population^8^, as activities in VR are more attractive and engaging for children than conventional therapy.

One of the principal groups of potential benefits from the application of VR training is patients with balance disorders, instability, and a high risk of falls. Among studies that evaluated the efficiency of VR in rehabilitation significant conclusions can be drawn from Parkinson's Disease studies, as this population suffers from balance impairments which significantly impact the quality of life. A recent meta-analysis of VR rehabilitation in Parkinson’s Disease included 14 randomized clinical trials and concluded, that the effects of VR therapy were most effective in improving balance compared to standard treatment^9^. Most of these studies use validated questionaires for the evaluation of symptoms and quality of life related to balance pre- and post- intervention. Studies utilizing objective, instrumentalized measures such as posturography are scarce. Newer VR systems are equipped with pressure sensors or gyroscopes that potentially allow to track posturographic parameters and compare them between interventions. In comparison to conventional balance exercises VR therapy has the advantage of being able to produce a “false” sense of environment, which can be either stable or dynamic. Furthermore, recent studies into the effect of immersive virtual reality on balance suggest, that the transient visual perturbations in VR boost short-term balance learning in virtual reality by modulating electrocortical activity^10^. Multiple systems have been utilized in VR-aided vestibular rehabilitation programs. Initially, these mostly comprised a pressure board or motion sensors and a flat-screen display^11,12^. Further development of screen technology enabled researchers to construct smaller dimension surround screen setups to perform balance tests on static posturography using VR stimuli^13^. However, the introduction of commercially available head-mounted displays (HMDs), such as the Oculus Rift in 2012, changed the perspective on Virtual Reality. The addition of HMDs has not only improved the feeling of immersion in virtual environments but also enabled researchers to design VR trials with a wider range of motion. However, a negative effect of increased immersion has also been observed—the use of HMDs produces motion intolerance symptoms referred to as cybersickness. Some studies found almost half of the participants could not complete a 10-min task in immersive Virtual Reality (VR)^14^.

The aging of the balance system can be the result of accumulative degenerative changes to primary sensory inputs—vestibular information can be altered or absent, somatosensory information can be distorted due to musculoskeletal disorders or reduced distal somatosation^15^, and finally, visual acuity may be compromised with age. Moreover, the central processing of this information may be impacted by central nervous system disorders, which also negatively impact balance^16^.

Recent developments in VR focus on delivering tele-neurorehabilitation programs to patients with balance disorders^17^. The introduction of such programs could reduce the economic strain of balance disorders and falls on healthcare systems by delivering individualized rehabilitation at home. However, the effect of age on the tolerance of VR has not been thoroughly assessed. So far, the literature reports one study that used an immersive virtual environment to measure the effect of age on balance in healthy individuals and a group of patients with bilateral vestibular loss^18^. To date, the influence of VR on sway parameters in movement has not been studied. As the overstimulation of older adults in VR could discourage them from participating in such programs by inducing cybersickness, we believe that the intensity of the VR experience should be consistent among a diverse group of patients.

This study aims to assess the effect of Virtual Reality on balance in an immersive virtual reality environment delivered by a head-mounted display and to establish the feasibility of using the head-mounted VR unit as a standalone posturography method. Our goal is to present a portable, low-cost solution for quantitive evaluation of balance in challenging situations, which could be used in clinical practice for the detection of patients in high risk of falls or general balance screening in everyday practice. The following research questions and hypotheses were formulated:

### Research question 1

Does an increase in visual perturbance delivered by an immersive virtual reality environment increase postural sway in a quiet stance?

### Hypothesis 1

Increasing disturbance of visual input by means of an immersive virtual reality environment increases postural sway.

### Research question 2

Is there a correlation between mobile posturography measurements and VR-headset posturography measurements?

### Hypothesis 2.

There is a correlation between lumbar mobile posturography angular velocity measurements and VR-headset-derived angular velocity measurements in static and walking posturography trials.

## Methods

### Ethics information

The research protocol was approved by the Bioethics Commission of the Medical University of Lodz (RNN/12/22/KE, 11.01.2022). All subjects provided written informed consent. All clinical investigations were conducted according to the principles expressed in the Declaration of Helsinki.

### Study design

The subjects for this study were recruited from a healthy population aged 18–80 years. Upon initial screening, the subjects underwent an otoneurologic examination that comprised an otoscopic examination and videonystagmography (VNG) testing, including a caloric test, optokinetic test, and saccadic pursuit. There was also a questionnaire that screened for current and past vestibular symptoms in the patients’ native language.

Inclusion criteria.

1. Ability to perform posturographic evaluations in VR.
2. Age 18–80 years

Exclusion criteria.

1. Any abnormalities in VNG suggestive of a central, mixed, or peripheral vestibular disorder.
2. Musculoskeletal or orthopedic pathology that, in the opinion of the investigators, would impact mobile posturography measurements.
3. A chronic hearing disorder including, but not limited to, hearing loss and tinnitus.
4. A history of VR intolerance
5. Indication of any vestibular disorder in the past in the screening questionnaire

**Figure 1:**
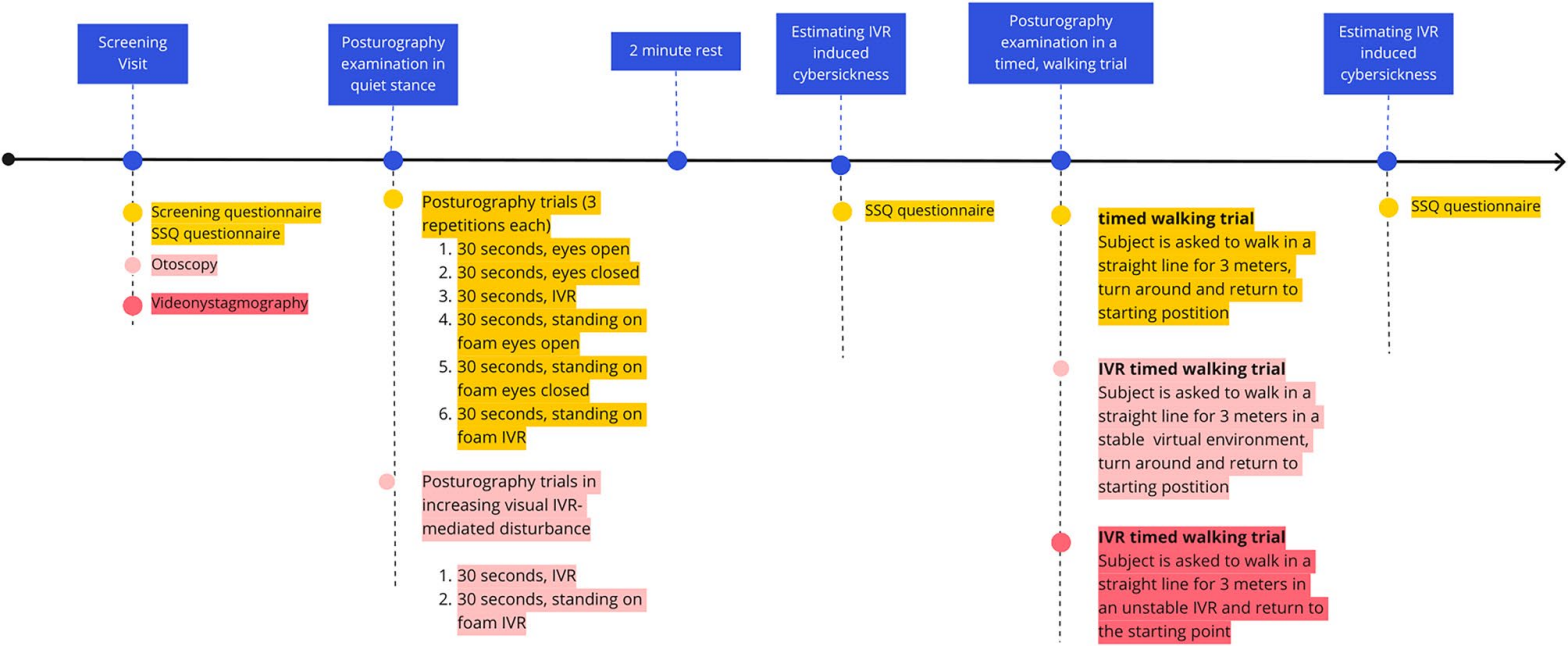
Study flowchart. IVR—Immersive Virtual Reality. SSQ—Simulator Sickness Questionnaire.

Participants who fulfilled the inclusion criteria and without any exclusion criteria were then assessed according to the design presented in Fig. 1.

The VR intervention was split into two segments to allow resting time for older adults and remove exertion interference on further evaluation.

The measurement system, tailored for research purposes, relies on two key electronic devices. The first is the Head Mounted Display (HMD), responsible for immersing the user in the artificial environment of the VR application. The Meta Quest 2 HMD kit is completely autonomous, meaning that it contains on board all the components necessary to operate the immersive application independently while accurately positioning the device in a specific room environment. The six-degrees-of-freedom (6DOF) motion tracking system integrates four-camera optical positioning relative to the user’s known environment, and its precision is augmented by additional sensors such as gyroscopes and accelerometers.

**Figure 2:**
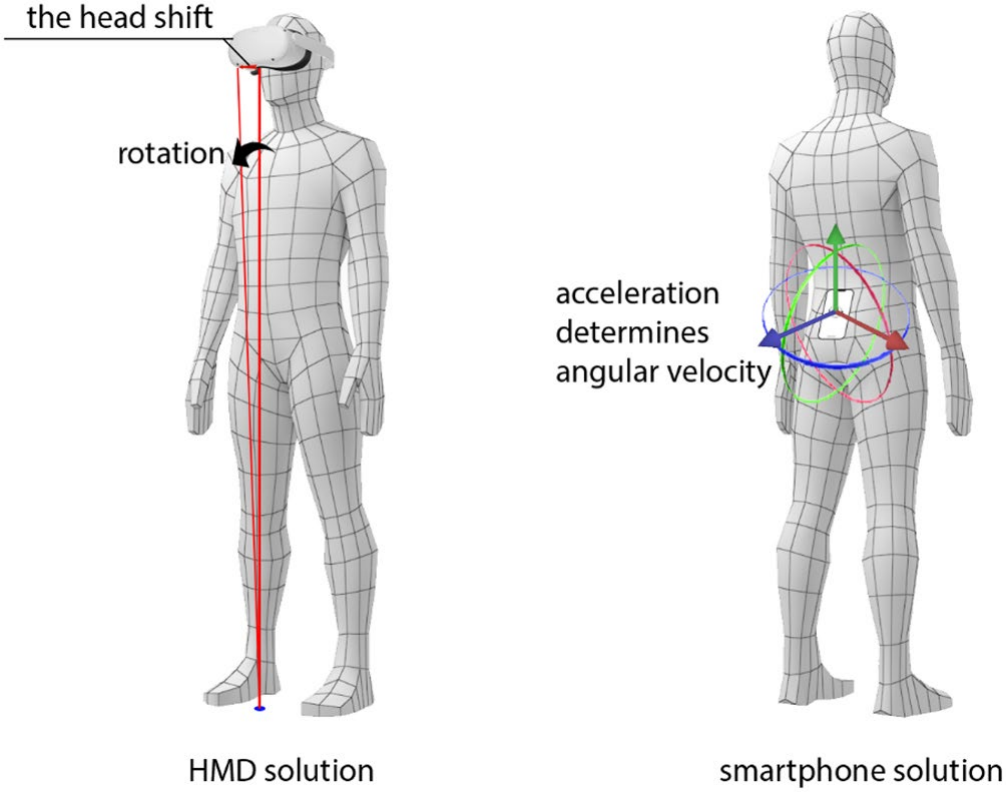
Conceptual illustrations of posturographic measurements utilizing HMD and smartphones.

The use of built-in HMD sensors enables the recording of user behaviors, interpreting them as posture indicators. These data encompass parameters such as angular velocity or the projection of the HMD’s position onto the floor plane, allowing an assessment of the user’s posture stability during tests (see Fig. 2 left side). The average projection of the user’s position in the first 3 s of measurement is a reference point for the angular velocity of the user’s silhouette. This method may be susceptible to parasitic or disruptive signals arising from the user’s head movements, which, despite provided instructions, can impact measurement precision.

The second device used in the research is a mid-range smartphone running on the Android operating system, equipped with standard built-in sensors such as a gyroscope, accelerometer, and compass. The device was mounted in the lumbar area and secured by an elastic strap (at waist height). Positioning inaccuracies were compensated for by the average direction of gravitational force determined in the first 3 s of the measurement session. Due to the impracticality of GPS in measurements, multi-axis acceleration sensor readings were solely used to determine angular velocities (see Fig. 2 right side).

**Figure 3:**
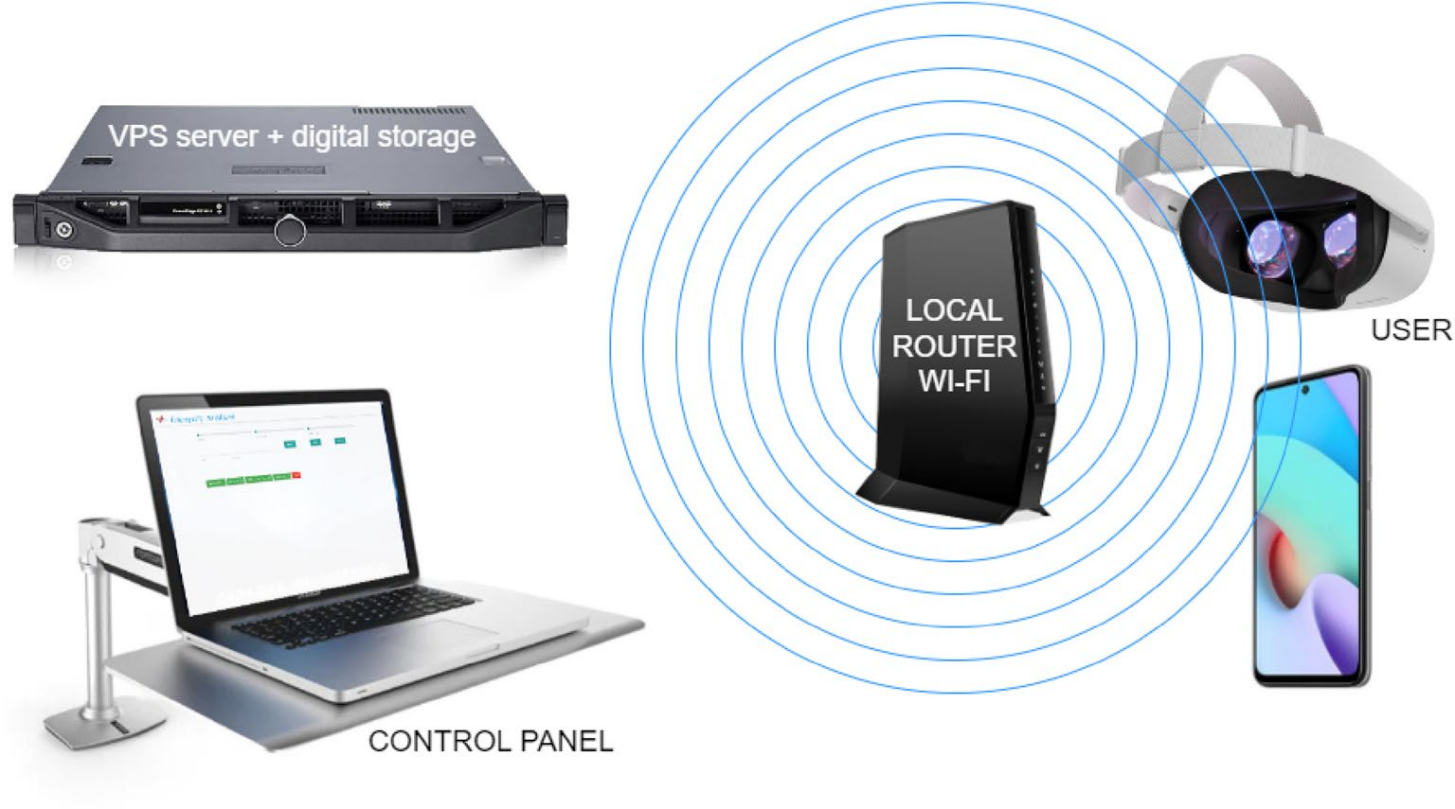
The architecture utilized for controlling and conducting the posturographic measurements.

Both devices were programmed using the Unity/C# development platform. In natural conditions, the system can record data with an interval of about 15 ms (67 Hz) for the HMD and about 30 ms (33 Hz) for the mobile phone. This was sufficient to precisely record the actual motion dynamics of the subjects during the research, as the sampling frequency significantly exceeded the frequencies of the observed “swaying” of patients. Measurement sessions commenced and concluded with an audible signal and, in the case of the mobile phone, an additional visual cue (for the conducting staff). The entire research procedure was remotely controlled through a control panel accessible via a standard web browser operated by the research personnel. Communication between the HMD/smartphone recording devices and the digital data storage repository occurred through a local Wi-Fi network (see Fig. 3). The results of each measurement session were buffered in the local resources of the devices and transmitted directly to an external digital data storage system upon completion. The application structure was tailored to the resources of a virtual VPS server with a Linux operating system and Node.js runtime environment.

Primary posturography evaluation was conducted following the modified Clinical Test of Sensory Interaction and Balance (mCTSIB). Additionally, a modification of the mCTSIB was performed where “eyes closed” trials had been replaced by a 30-s trial in a head-mounted Virtual Reality device (Oculus Quest 2, Meta Technologies LLC, USA).

The VR environment of the experiment was in the form of a medium-sized transportation ship floating in the open sea. The situation the users find themselves in is simulated as accurately as possible—the ship is surrounded by water, and the clear horizon line is visible, which fully justifies the tilt effect.The first segment of the VR trial focused on delivering altered visual information in a quiet stance. The measurement procedure was initiated after the patient had been correctly positioned in the marked area, which was confirmed by visual and acoustic signals (a green light in the upper part of the screen/view in HMD). The researcher used an application synchronized with the HMD, which could alter the tilt, period, and sensitivity of the motion of the ship on waves ranging from 0 (no perception of movement) to 70 (perception of a heavy storm).

Each trial (1–4) was repeated three times, and an average value was calculated. If the patient fell and a trial was interrupted, this measurement was labeled as a fall and re-taken.

**Figure 4:**
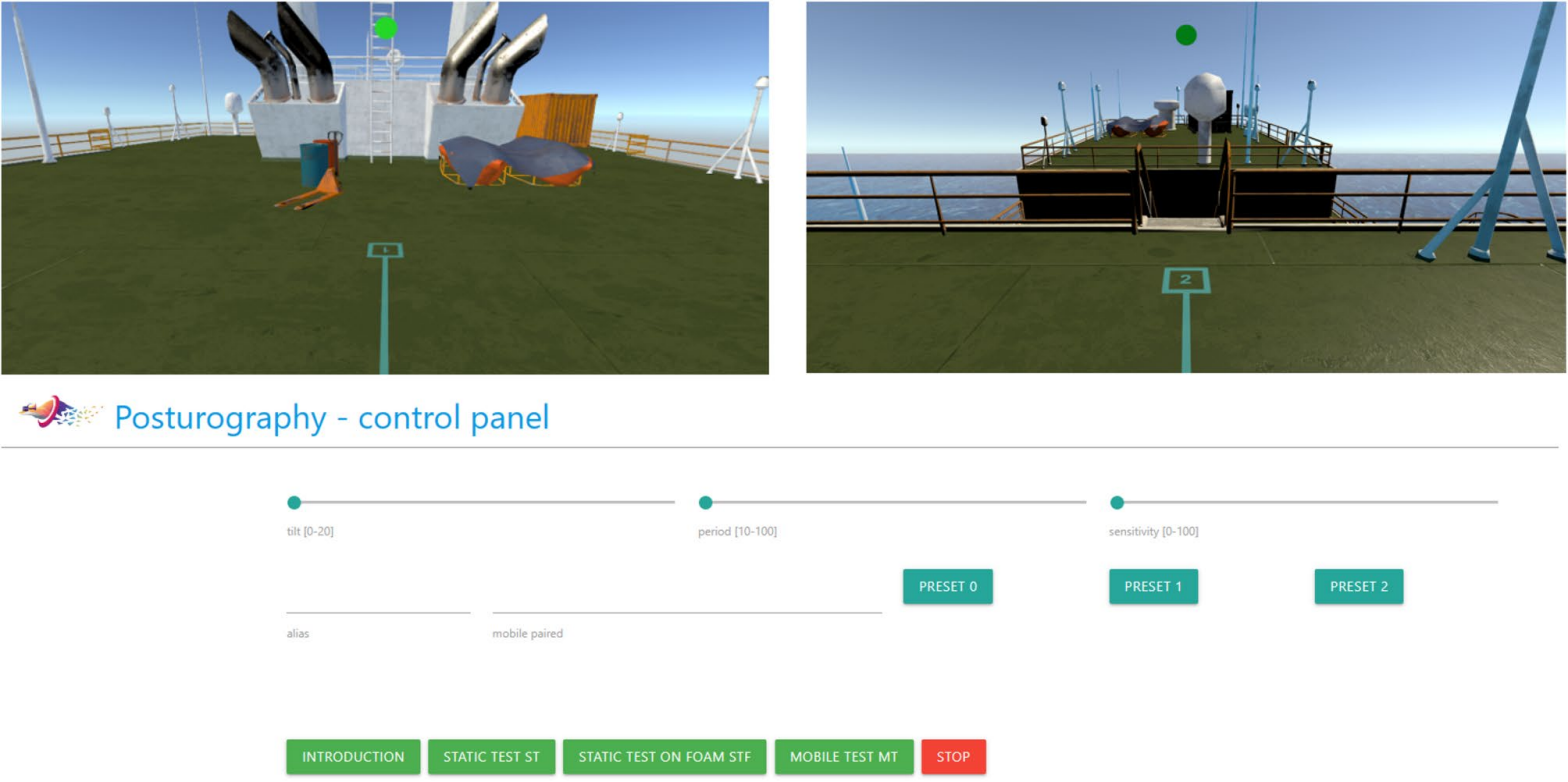
Immersive Virtual Reality environment and control panel.

The second trial was attempted after the rest period. The patient stood on a firm surface and was asked to walk without any VR in a straight 3-m line marked with a visible line on the examination room floor, turn around at the end of the line, and return to the starting point. The time to complete the trial was measured simultaneously with mobile posturography measurements from a lumbar-mounted sensor. Mobile posturography was used to calculate the mean angular velocity during this trial. The subject was then equipped with head-mounted VR and asked to perform a similar task in the stable VR environment of the cargo ship, where a line on the virtual floor connected the starting position to a second point. After starting the procedure (researcher panel/start button), the patient was informed about the start of the procedure acoustically and visually 3 s in advance. The patient was informed that they should proceed on foot in a straight line to the next location (marked [2]) 3 m away physically. After covering a distance of 3 m +/− 0.3 m, they were asked to turn 180 degrees and return to the starting point (marked [1]). These markings are visible in Fig. 4. Once the target was reached, the measurement procedure stopped. The measurement procedure was limited to 30 s; if the time was exceeded, the measurement procedure was automatically stopped, and the patient was informed about it acoustically and visually). The Last VR trial was conducted similarly, but the visual environment was rendered unstable and represented a slowly swaying surface of a ship using parameters accessible through the researcher panel, similar to static trials.

After completing both stages of the posturographic measurements, the participant was asked to complete a Simulator Sickness Questionnaire (SSQ), which had been previously translated and validated in the Polish language by Biernacki et al.^19^.

### Statistical analysis

To measure test–retest reliability, the intra-class correlation coefficient (two-way, mixed effects, single rater, absolute agreement) was calculated as per the guideline described by Koo et al.^20^ Inter-rater reliability between the two posturography methods for consecutive, simultaneous measurements was assessed using inter-class correlation coefficient (two-way, mixed effects, mean of k raters, absolute agreement). Continuous variables were tested for normality using the Shapiro–Wilk test, and for non-normal distribution, median values and interquartile ranges were provided. These variables were analyzed using non-parametric tests. When 3 or more series were analyzed, we used Friedman ANOVA. The level of significance used for all analyses was 2-tailed and set at *p* < 0.05. Statistical analysis was performed using STATISTICA software (Version 13.1, Dell).

## Results

### Study population

Thirty-eight patients fulfilled the inclusion criteria and none of the exclusion criteria and were included in the study. The screening examination did not reveal any vestibular or auditory dysfunction upon clinical examination. The youngest participant was 19 years old; the oldest participant was 76 years old. The median age was 34 years. The male-to-female ratio was 17:21. The average weight of participants was 75 kg (SD:19.06), while the average height was 167.3 cm (SD:7.79).

### Reliability

The test–retest reliability of the two methods of measuring angular velocity was evaluated using the inter-class correlation coefficient (two-way, mixed effects, single rater, absolute agreement). The ICC was then interpreted following Koo et al., where values less than 0.5, between 0.5 and 0.75, between 0.75 and 0.9, and greater than 0.9 are indicative of poor, moderate, good, and excellent reliability, respectively.

For the VR headset, the ICC at static conditions was good to excellent regardless of the intensity of the VR environment. The ICC was significantly lower for the 3-m walking trials, with values from 0.527 to 0.713 indicative of poor to moderate reliability.

The smartphone-derived measurement presented significantly worse test–retest capabilities with the ICCs in static conditions, ranging from 0.522 to 0.749, indicative of poor to moderate reliability; the 3-m walking trial measurement ICCs were estimated at 0.355, indicative of poor reliability. The ICC values for all trials are provided in Table 1.

**Table 1:** Test–retest parameters derived from posturography trials from mobile posturography and head-mounted display. Inter-class correlation coefficient values are provided.

Measurement device	Trial	No VR	VR stable	VR waves	VR storm
VR Posturography (location—head)	3-m walking trial	NA	0.692	0.839	0.713
	Stable surface	NA	0.862	0.825	0.886
	Foam	NA	0.918	0.972	0.898
Mobile posturography (location—lumbar area)	3-m walking trial	0.355	0.487	0.512	0.448
	Stable surface	0.749	0.782	0.684	0.673
	Foam	0.522	0.694	0.604	0.659

*NA* not applicable, *VR* virtual reality.

### Effect of VR on static posturography trials

**Figure 5:**
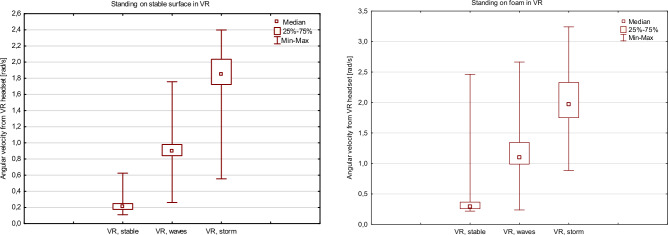
Box and whisker plots representing the influence of the changing VR environment on sway velocity measured from the head-mounted device (VR headset).

**Figure 6:**
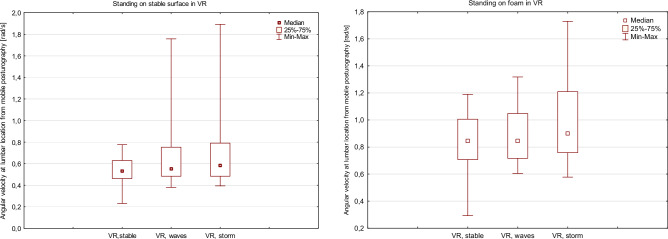
Box and whisker plots representing the influence of the changing VR environment on sway velocity measured from the lumbar-mounted device (mobile posturography).

All patients completed static trials. A significant difference was observed between the measurements at the lumbar area and those derived from the headset. We observed a strong influence of the visual disturbance caused by increasing wave frequency and tilt in the VR environment. Initial angular velocity at head level increased 4.2 times in the trials on a stable surface with mild waves at sea and 8.8 times at storm at sea. This effect was statistically significant (Friedman ANOVA *p* < 0.001). Similar observations were made for standing on foam trials where a 3.79-fold increase was observed for mild conditions and a 6.7-fold increase for storm at sea conditions (Friedman ANOVA *p* < 0.001). This is represented in Fig. 5. We did not observe any difference in angular velocity measured at the lumbar area as seen in Fig. 6. This is also summarized in Table 2.

**Table 2:** Median angular velocity values from the Virtual Reality headset measured during stance on a stable surface and foam. All values are the average of at least two successful attempts at the trial.

Measurement device	Trial	No VR, eyes open	No VR, eyes closed	VR, stable environment (median angular velocity rad/s, IQR)	VR, mild waves at sea (median angular velocity rad/s, IQR)	VR, storm at sea (median angular velocity rad/s, IQR)
VR headset (location—head)	Quiet stance	NA	NA	0.212 (0.072)	0.899 (0.138)	1.850 (0.312)
	Standing on foam	NA	NA	0.294 (0.108)	1.099 (0.355)	1.967 (0.578)
Smartphone (location—lumbar area)	Quiet stance	0.533 (0.333)	0.565 (0.174)	0.533 (0.167)	0.549 (0.268)	0.583 (0.308)
	Standing on foam	0.846 (0.338)	1.074 (0.462)	0.846 (0.298)	0.845 (0.331)	0.901 (0.451)

*IQR* inter quartile range, *NA* not applicable (VR headset could not be used), *VR* virtual reality.

### Effect of VR on walking trials

**Figure 7:**
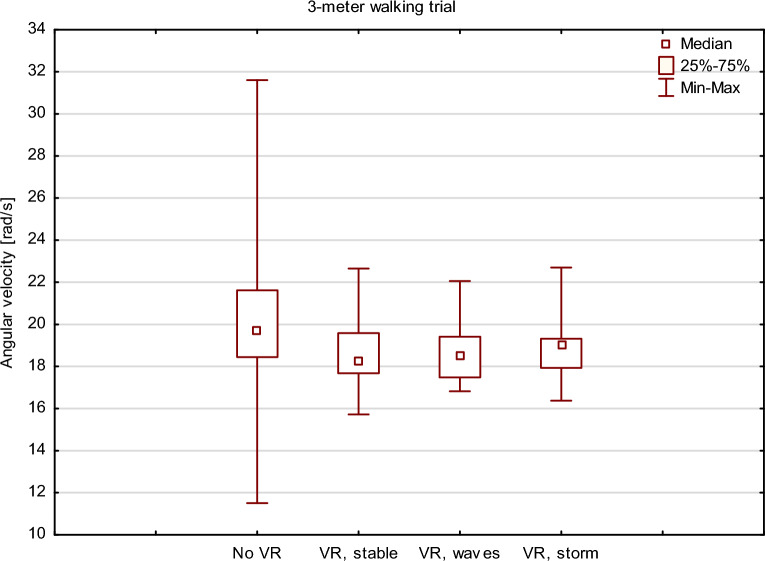
Box and whisker plots representing the influence of the changing VR environment on angular velocity measured from the lumbar mounted device (mobile posturography) during the 3-m walking trial.

All patients completed the series of 3-m walking trials. In subjective evaluation after the examination, the patients found the VR trials in the storm environment more challenging. However, this is not reflected in the results of the mobile posturography measurements. Friedman ANOVA and post-hoc testing revealed a significant difference in angular velocity between the 3-m walking trial without any VR environment and the VR-assisted trials (Friedman ANOVA *p* = 0.013), but there were no significant changes in angular velocity while performing these tasks at increasing VR visual perturbance. This is shown in Fig. 7.

### Evaluation of cybersickness induction

**Figure 8:**
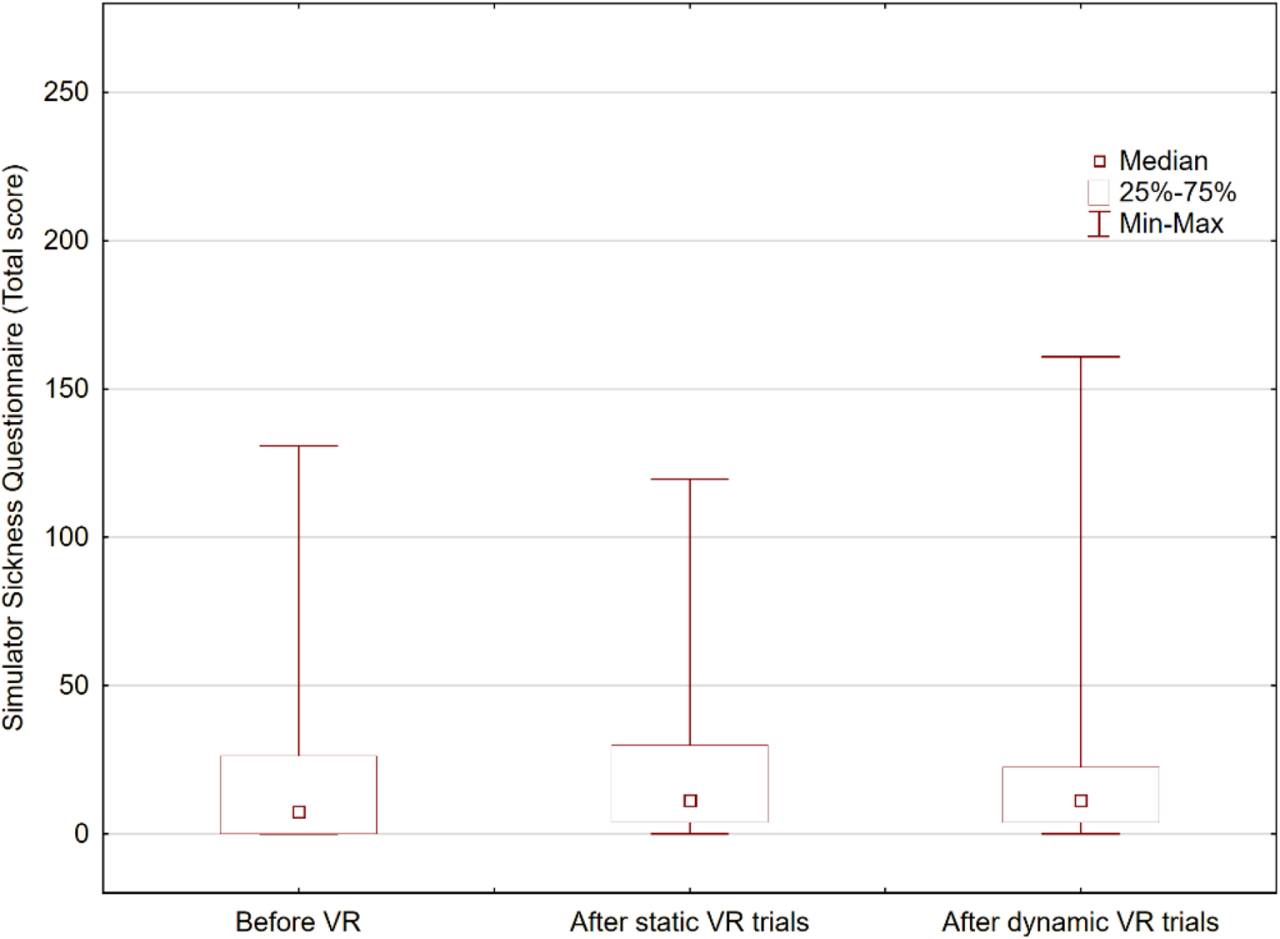
Box and whisker plots representing the total Simulator Sickness Questionnaire score at initial evaluation and after consecutive trials in virtual reality.

In subjective evaluation, two patients (5%) reported significant side effects of the virtual reality environment; they were able to complete the trial without interruption. The median SSQ before the application of VR was 7.48 (IQR 18.7), and after static trials in VR, the median SSQ score was 11.22 (IQR 24.31). After the walking VR trials, the median SSQ score was 11.22 (IQR 18.7). No significant differences were observed (Friedman ANOVA p = 0.495). The results of SSQ are shown in Fig. 8. For patients who reported cybersickness symptoms, the initial evaluation was 0 points for both patients; after the static VR trials, the SSQ total for patient 1 was 29.92, and for patient 2 it was 119.68. After the walking VR trial, this further increased for patient 1 to 160.82 and for patient 2 to 123.42. The maximum total score of the SSQ in the Polish translation is estimated at 291.72 points. These patients complained of autonomic symptoms, mainly nausea, sweating, salivation, and headache.

Correlation between head and lumbar measurements.

The correlation between head and lumbar measurements derived from the two different devices was evaluated using the Spearman rank correlation. For the 3-m walking trials, no significant correlations were found. For the static trials, there was only one clinically and statistically significant correlation between head-derived measurement and lumbar-derived measurement for VR in storm conditions (r = − 0.607), indicative of a strong negative relationship. The correlogram is included as Supplementary Table 1.

## Discussion

In this study, we evaluated the influence of the VR environment on the sway parameters of a healthy population. As a reference to other studies on balance, we chose mobile posturography at the lumbar area as the standard methodology, while the head-derived measurement was investigated. The study proves a significant effect of the increasing visual disturbance in the virtual environment on sway parameters. We chose angular velocity as the reference parameter, as in our previous research regarding wearable sensors and accelerometry^21^. We found that a static and stable virtual environment has little influence on angular velocity. A recent systematic review of the influence of VR head-mounted displays on balance outcomes by Soltani et al.^22^ pointed out that only one study exists that evaluated the validity and reliability of HMD systems for assessing balance. Alahmari et al. in 2014^23^ compared VR-assisted dynamic posturography BRU (Balance Rehabilitation Unit, Interacoustics, Denmark) with conventional dynamic posturography. For this comparison, they used the Center of Pressure (COP) parameters, which are derived from force-plate sensors and the Sensory Organization Test result. Alahmari et al. found the COP area and sway velocity correlated with the measurement of the SOT (Sensory Organization Test, an inbuilt measure of BRU Dynamic Posturography).

In our previous study, we compared the COP measurements and Center of Mass measurements derived from inertial sensors placed at the lumbar area. We found these values to be correlated but not directly comparable^21^. In this study, we have attempted to evaluate sway parameters directly from the VR headset to assess its feasibility as a standalone posturographic measurement. The abovementioned systematic review also mentions one study that addressed the reliability of VR HMD to assess balance and used force-plate posturography as a reference. Saldana et al. evaluated two groups of patients (healthy volunteers and patients at risk of falls) using simultaneous measurement of sway with a VR HMD and a static posturography measurement. They measured the reliability of VR posturography by calculating the correlation between measurements taken 1 week apart and judged the VR measurement to be highly reliable. The research protocol included trials on stable surfaces and foam, similar to our design. They concluded that all trials were able to detect differences in sway between eyes closed and stable surface conditions. In our trial, we also observed statistically significant differences between eyes open and stable surface and other trials; however, our analysis was restricted to angular velocity.

Saldana et al. also highlighted that VR HMD measuring points cannot be converted to approximate the position of the center of mass. In our study, we found no significant correlation between mobile posturography at the lumbar level (approximate center-of-mass measurement) and VR HMD posturography measurements, which provides further proof that the two methodologies are not directly related. This can be explained by the movement strategies employed to maintain balance. The ankle strategy, where the body moves at the ankle according to the inverted pendulum model, is utilized to maintain balance for small amounts of sway and on a stable surface. The hip strategy, used for greater sway disturbances, uses the hips to quickly react to sudden displacements of the center of mass on unsteady surfaces^24^. We observed that the movements of the head and hip during trials on unstable surfaces or when the visual input was altered in VR were asynchronous. Additionally, the tilt at the head level was greater than at the lumbar level, which, in our opinion, accounts for the lack of correlation between those measurements. The two measures are not correlated and directly comparable. In our sample, we observed better reliability for VR HMD measurements than for mobile posturography, which can be attributed to the higher resolution of inertial sensors used in commercially available VR devices.

One of the disadvantages of immersive VR environments is the possibility of inducing cybersickness symptoms. Occluding peripheral vision during VR experience is known to induce cybersickness symptoms; however, retaining external peripheral vision reduces immersion from the virtual experience^23^. Another factor that contributes to motion intolerance in VR is rotation along the longitudinal axis, which is often limited in modern VR applications to mitigate cybersickness. In our study, only one task included rotation along the longitudinal axis—the 3-m walking test in VR, where at the end of the line, the patient was asked to make a 360-degree turn and return to the starting position. In our pre- and post-exposure assessments, we did not observe significant side effects measured by the SSQ questionnaire. Only two patients complained of cybersickness symptoms, of whom only one reported an increase in symptoms in the second evaluation, after the walking trials. Saldana et al. reported one patient who, due to cybersickness symptoms, did not report for a second visit. Our patients completed the assessment, although in our trial, there was only one VR session. One of our participants stated that due to the cybersickness symptoms, he would not like to repeat this experience. Only 5% of participants complained of side effects, while the remaining participants experienced negligible effects of motion intolerance, which is reflected in the mean difference of SSQ 7.89 (SD: 37.44 [95% CI 30.37;48.84]). Furthermore, both patients were from the younger group of participants (24 and 30 years old), which is contrary to the popular belief that VR is tolerated worse with age. Overall, the VR HMD assessment was well tolerated despite the additional visual stimulus of bouncing on sea waves that we employed in our study.

In this study, the VR headset was not capable of eye-tracking therefore no information is available on the eye-movement, vestibulo-ocular reflex or eye movement patterns. Recently introduced VR headsets such as the Oculus Meta Quest Pro (Meta Technologies LLC, USA ) or Apple Vision Pro (Apple Inc., USA) are equipped with infrared cameras that track eye movement similarly to the cameras in videonystagmography googles^25^. Future studies could include monitoring eye-movement patterns through such cameras as adding an additional biosignal might improve the overall sensitivity and specificity of the proposed solution. Furthermore, integration of eye-trakicng and artificial intelligence into the VR software could aid in monitoring of patients with neurodegenerative diseases^26^.

The spatial information that is necessary to compute complex VR environments and transfer precise movements of the user to the environment in modern VR headsets is very accurate. Commercially available VR HMDs may induce vestibulo-ocular conflict and elicit vestibulo-spinal reflexes that can be measured by either standalone posturography or directly from the inertial sensors. Immersive VR can place patients in different visual and situational scenarios, which could enhance the diagnostic yield of posturography in differentiating people with balance impairments and risk of falls from healthy population. Further studies are required to establish cut-off values for balance-impaired populations and the reliability of measurements in people with various vestibular disorders. After expanding the study into balance-impaired populations we believe that our solution could be used as a low-cost screening application to detect people at high risk of falls in a more efficient manner than conventional tests. This solution might be particularly appealing to general practitioners, occupational medicine specialists or physiotherapists specializing in the rehabilitation of balance to screen for patients requiring interventions to reduce the risk of falls or those requiring further, more sophisticated vestibular testing.

### Limitations

This was an exploratory study in a healthy population to establish the reliability of balance testing by VR HMDs as standalone devices; in the future, various populations with balance disorders should be examined. We will use the data acquired in this pilot study in phase two of our project to determine the cut-off values and diagnostic accuracy of this methodology and compare it with standard posturography. The participants in this study were fully capable of performing our VR trials, but safety mechanisms should be implemented for patients with motor impairments or severe vestibular dysfunction. The VR system software can currently represent the boundaries of the virtual environment. As the patient approaches the predetermined boundary, a virtual safety net is displayed to prevent collisions with objects. Other solutions could include a suspension harness, but the interference of such a solution on posturography measurements merits further study. For severely motor-impaired patients, a seated evaluation in a VR headset is possible. However, a direct comparison of posturography parameters with an assessment in a standing position requires a separate study.In our group only four participants had any previous exposure to immersive VR, and this did not account for any statistically significant differences in posturographic trials. However, the effect of adaptation to VR on balance under changing visual conditions should be separately studied to fully account for this possible confounding factor.

## Conclusions

Immersive Virtual Reality delivered through head-mounted displays influences sway velocity measured at head level. Increasing the disturbance of visual information in VR causes an increase in sway parameters. A modern, commercially available VR HMD is capable of reliable and valid sway measurements and could serve as a standalone posturography device. No correlation was found between the center of mass velocity measurements from mobile posturography and head velocity measurements from HMD. This method is well tolerated and has a low risk of inducing cybersickness symptoms.

### Supplementary Information

Supplementary Information.

## Data Availability

The datasets generated during and/or analyzed during the current study are available from the corresponding author upon reasonable request.
