# Supplementary material for: The effect of Immersive Virtual Reality on balance: an exploratory study on the feasibility of head-mounted displays for balance evaluation
Source: Sci Rep. 2024 Feb 12;14:3481. doi: 10.1038/s41598-024-54274-8 (PMC10861529; doi:10.1038/s41598-024-54274-8)
Supplement: Supplementary file 1 — Supplementary Information. [file 41598_2024_54274_MOESM1_ESM.docx]

| Supplementary Table 1 – Spearman rank correlation coefficient matrix of angular velocity values measured from head-mounted sensor (VR) and lumbar sensor  (mobile posturography) at different trials |
| --- |
| \| VRSTFP1  MOBILE \| 1 \| 0,576 P=0,0040 \| 0,815 P<0,0001 \| 0,232 P=0,2863 \| 0,415 P=0,0489 \| 0,300 P=0,1637 \| 0,396 P=0,0612 \| -0,194 P=0,4125 \| -0,128 P=0,5697 \| -0,281 P=0,2059 \| 0,411 P=0,0513 \| -0,619 P=0,0047 \| \| --- \| --- \| --- \| --- \| --- \| --- \| --- \| --- \| --- \| --- \| --- \| --- \| --- \| \| VRSTP2  MOBILE \| 0,576 P=0,0040 \| 1 \| 0,610 P=0,0026 \| 0,671 P=0,0005 \| 0,359 P=0,0928 \| -0,067 P=0,7607 \| -0,056 P=0,7985 \| -0,224 P=0,3423 \| 0,091 P=0,6874 \| -0,037 P=0,8712 \| 0,257 P=0,2366 \| -0,430 P=0,0663 \| \| VRSTFP2  MOBILE \| 0,815 P<0,0001 \| 0,610 P=0,0026 \| 1 \| 0,483 P=0,0229 \| 0,382 P=0,0791 \| 0,129 P=0,5663 \| 0,233 P=0,2963 \| -0,053 P=0,8306 \| -0,044 P=0,8493 \| -0,238 P=0,2996 \| 0,214 P=0,3389 \| -0,607 P=0,0059 \| \| VRSTP1  MOBILE \| 0,232 P=0,2863 \| 0,671 P=0,0005 \| 0,483 P=0,0229 \| 1 \| 0,321 P=0,1351 \| -0,004 P=0,9857 \| -0,289 P=0,1818 \| -0,123 P=0,6045 \| 0,181 P=0,4195 \| 0,018 P=0,9384 \| 0,030 P=0,8932 \| -0,175 P=0,4725 \| \| VRSTP0 \| 0,415 P=0,0489 \| 0,359 P=0,0928 \| 0,382 P=0,0791 \| 0,321 P=0,1351 \| 1 \| 0,299 P=0,1652 \| -0,262 P=0,2274 \| -0,002 P=0,9934 \| -0,214 P=0,2643 \| 0,083 P=0,6738 \| 0,401 P=0,0282 \| -0,329 P=0,1013 \| \| VRSTP0  MOBILE \| 0,300 P=0,1637 \| -0,067 P=0,7607 \| 0,129 P=0,5663 \| -0,004 P=0,9857 \| 0,299 P=0,1652 \| 1 \| 0,409 P=0,0526 \| 0,266 P=0,2567 \| -0,220 P=0,3260 \| 0,037 P=0,8712 \| -0,033 P=0,8826 \| 0,075 P=0,7589 \| \| VRSTFP0  MOBILE \| 0,396 P=0,0612 \| -0,056 P=0,7985 \| 0,233 P=0,2963 \| -0,289 P=0,1818 \| -0,262 P=0,2274 \| 0,409 P=0,0526 \| 1 \| 0,311 P=0,1816 \| -0,037 P=0,8712 \| -0,174 P=0,4374 \| 0,113 P=0,6088 \| -0,070 P=0,7753 \| \| VRSTP2 \| -0,194 P=0,4125 \| -0,224 P=0,3423 \| -0,053 P=0,8306 \| -0,123 P=0,6045 \| -0,002 P=0,9934 \| 0,266 P=0,2567 \| 0,311 P=0,1816 \| 1 \| 0,240 P=0,2479 \| 0,279 P=0,1670 \| -0,263 P=0,1944 \| 0,393 P=0,0574 \| \| VRSTP1 \| -0,128 P=0,5697 \| 0,091 P=0,6874 \| -0,044 P=0,8493 \| 0,181 P=0,4195 \| -0,214 P=0,2643 \| -0,220 P=0,3260 \| -0,037 P=0,8712 \| 0,240 P=0,2479 \| 1 \| 0,045 P=0,8253 \| -0,098 P=0,6147 \| 0,094 P=0,6555 \| \| VRSTFP1 \| -0,281 P=0,2059 \| -0,037 P=0,8712 \| -0,238 P=0,2996 \| 0,018 P=0,9384 \| 0,083 P=0,6738 \| 0,037 P=0,8712 \| -0,174 P=0,4374 \| 0,279 P=0,1670 \| 0,045 P=0,8253 \| 1 \| -0,435 P=0,0207 \| 0,498 P=0,0112 \| \| VRSTFP0 \| 0,411 P=0,0513 \| 0,257 P=0,2366 \| 0,214 P=0,3389 \| 0,030 P=0,8932 \| 0,401 P=0,0282 \| -0,033 P=0,8826 \| 0,113 P=0,6088 \| -0,263 P=0,1944 \| -0,098 P=0,6147 \| -0,435 P=0,0207 \| 1 \| -0,506 P=0,0084 \| \| VRSTFP2 \| -0,619 P=0,0047 \| -0,430 P=0,0663 \| -0,607 P=0,0059 \| -0,175 P=0,4725 \| -0,329 P=0,1013 \| 0,075 P=0,7589 \| -0,070 P=0,7753 \| 0,393 P=0,0574 \| 0,094 P=0,6555 \| 0,498 P=0,0112 \| -0,506 P=0,0084 \| 1 \| \|  \| VRSTFP1  MOBILE \| VRSTP2  MOBILE \| VRSTFP2  MOBILE \| VRSTP1  MOBILE \| VRSTP0 \| VRSTP0  MOBILE \| VRSTFP0  MOBILE \| VRSTP2 \| VRSTP1 \| VRSTFP1 \| VRSTFP0 \| VRSTFP2 \| |

VRSTP0 - Head-mounted sensor, quiet stance in virtual reality, stable environment, VRSTP1- Head-mounted sensor, quiet stance in virtual reality, unstable environment – waves, VRSTP2- Head-mounted sensor, quiet stance in virtual reality, unstable environment – storm, VRSTFP0 - Head-mounted sensor, stance on foam in virtual reality, stable environment, VRSTFP1- Head-mounted sensor, stance on foam in virtual reality, unstable environment – waves, VTSTFP2 - Head-mounted sensor, stance on foam in virtual reality, unstable environment – storm,VRSTPMOBILEP0- lumbar sensor, quiet stance in virtual reality, stable environment, VRSTPMOBILEP1 - lumbar sensor, quiet stance in virtual reality, unstable environment – waves, VRSTPMOBILEP2 - lumbar sensor, quiet stance in virtual reality, unstable environment – storm, VRSTPMOBILEP0 - lumbar sensor, stance on foam in virtual reality, stable environment, VRSTFPMOBILEP1 - lumbar sensor, stance on foam in virtual reality, unstable environment – waves, VRSTFPMOBILEP2 - lumbar sensor, stance on foam in virtual reality, unstable environment - storm
